# Bismuth Nanoantibiotics Display Anticandidal Activity and Disrupt the Biofilm and Cell Morphology of the Emergent Pathogenic Yeast *Candida auris*

**DOI:** 10.3390/antibiotics9080461

**Published:** 2020-07-29

**Authors:** Roberto Vazquez-Munoz, Fernando D. Lopez, Jose L. Lopez-Ribot

**Affiliations:** 1 Department of Biology, and South Texas Center for Emerging Infectious Diseases, The University of Texas at San Antonio, San Antonio, TX 78249, USA; 2 School of Engineering, The University of Texas at Austin, Austin, TX 78712, USA; fernandod.lopez2016@gmail.com

**Keywords:** nanoantibiotics, *Candida auris*, biofilms, pathogenic fungi, bismuth nanoparticles, novel agents, multidrug-resistance, SEM analysis, biofilm structure

## Abstract

*Candida auris* is an emergent multidrug-resistant pathogenic yeast, which forms biofilms resistant to antifungals, sanitizing procedures, and harsh environmental conditions. Antimicrobial nanomaterials represent an alternative to reduce the spread of pathogens—including yeasts—regardless of their drug-resistant profile. Here we have assessed the antimicrobial activity of easy-to-synthesize bismuth nanoparticles (BiNPs) against the emergent multidrug-resistant yeast *Candida auris*, under both planktonic and biofilm growing conditions. Additionally, we have examined the effect of these BiNPs on cell morphology and biofilm structure. Under planktonic conditions, BiNPs MIC values ranged from 1 to 4 µg mL^−1^ against multiple *C. auris* strains tested, including representatives of all different clades. Regarding the inhibition of biofilm formation, the calculated BiNPs IC_50_ values ranged from 5.1 to 113.1 µg mL^−1^. Scanning electron microscopy (SEM) observations indicated that BiNPs disrupted the *C. auris* cell morphology and the structure of the biofilms. In conclusion, BiNPs displayed strong antifungal activity against all strains of *C. auris* under planktonic conditions, but moderate activity against biofilm growth. BiNPs may potentially contribute to reducing the spread of *C. auris* strains at healthcare facilities, as sanitizers and future potential treatments. More research on the antimicrobial activity of BiNPs is warranted.

## 1. Introduction

Infectious diseases are among the first causes of death worldwide [1] and pose a burden for global health and economy, as they negatively impact major social aspects of everyday life [2]. The main threats associated with communicable diseases are the rise of drug-resistance, the limited number and diversity of available treatments, and the emergence of new pathogens [3,4,5], some of them with pandemic potential. The Candida genus is the main cause of fungal diseases [6,7]. Every year, more than 250,000 people worldwide are affected by invasive candidiasis, leading to more than 50,000 deaths, as the mortality can be as high as 40%, even when patients receive antifungal therapy [6]. *Candida albicans* is the main cause of Candida-related diseases; however, other *Candida* species also represent a health risk [8]. Among them, *C. auris*, an emergent pathogenic yeast, has risen as a clinical concern worldwide, as it spreads in healthcare-related facilities and has caused several outbreaks around the world [9,10].

*C. auris* is an ovoid-shaped yeast which has been described to be non-dimorphic, yet, some strains can display the pseudohyphae shape [11]. This yeast has been classified as an urgent threat by the CDC, as it has displayed resistance to different antifungal drug classes (multidrug-resistant) [3,12]. *C. auris* causes invasive infections with high mortality rates, particularly for inpatient care for patients that stay hospitalized for a long time or who are subject to surgery or intubation procedures [11,13]. Since its first description in 2009, it has rapidly spread around the globe. The different *C. auris* strains are classified in four geographic clades that may have emerged independently, and almost simultaneously, in different regions: South Asian (clade I), East Asian (clade II), South African (clade III), and South American (clade IV) [10,14]. Recently, in Iran, a potential fifth clade has been identified [15]. Each clade displays distinctive genotypic (genetic polymorphisms) and phenotypic traits (morphology, metabolism, and pathogenicity, among others), although the differences between the clades are minimal [10]. Additionally, there are differences regarding their drug-susceptibility profile [16] and behavior, as some can form aggregates [17].

*C. auris* inherent multidrug-resistance and its ability to form very resistant biofilms challenge our ability to reduce its spread [9,18]. This yeast is known to be resistant to sanitizing procedures [19] and to the main classes of antifungal drugs, particularly to fluconazole [16,20], which is among the most used anticandidal drugs, alongside with amphotericin B and echinocandins [21]. The biofilm stage allows *C. auris* to colonize and survive in different surfaces in healthcare facilities for long periods [13], under unfavorable environments, such as high temperature and salinity [22,23]. Furthermore, *Candida* biofilms can colonize implantable biomedical devices [24] and other medical instrumentation, which increases the risk in healthcare facilities worldwide. As *C. auris* displays enhanced resistance to current antifungal drugs and sanitizers, novel treatments are needed to effectively combat this pathogenic yeast. Currently, new anticandidal treatments are being researched, such as antifungal peptides [25], drug repurposing [26], and new antifungal drugs [27]. Recently, nanotechnology has been studied to combat *C. auris* [28,29].

Nanomaterials are an emergent alternative to combat infectious diseases. While numerous nanotechnology-based drugs (nanopharmaceuticals) are currently under preclinical and clinical development [30,31], many are now available for use in healthcare, such as vaccines and nanoantibiotics [32,33,34]. Nanomaterials display a wide range of inhibitory activities against a broad diversity of pathogens—including viruses, bacteria, fungi, and protozoa—and can even overcome their resistance to current antimicrobial drugs [35,36,37,38,39]. The use of antimicrobial nanomaterials (nanoantibiotics) is a novel approach to combat *C. auris*, due to the possibility of synthesizing cost-effective, potent disinfectants and treatments. The antifungal activity of nanoantibiotics on *C. auris* is virtually unknown; however, their anticandidal effect has been widely demonstrated in the literature [40,41,42,43,44,45,46]. More recently, our group demonstrated that AgNPs are effective against *C. auris* biofilms [29,47].

For this study, we evaluated the inhibitory activity of bismuth nanoparticles (BiNPs) against *C. auris*. Bismuth compounds have been historically used as antimicrobial agents [48], and some bismuth-based compounds and BiNPs exhibit anticandidal activity against *C. albicans* [49,50]. In a recent report, we described a new method for producing PVP-coated BiNPs with potential antimicrobial activity [51] and subsequently demonstrated their antibacterial and antifungal activity against *Staphylococcus aureus* and *C. albicans*, respectively [52]. In this work, we hypothesized that BiNPs display anticandidal activity against the different strains of *C. auris*. Our aims were to determine the inhibitory effect on the planktonic and the biofilm stages on different strains from the known clades of *C. auris* and then to assess the influence of nanoparticles on the phenotype and structural organization of the biofilms. In Appendix A, concepts such as planktonic and biofilm stages, and pseudohyphae are described.

## 2. Results and Discussion

The antifungal activity of the BiNPs was determined on the planktonic and biofilms stages of different strains representing multiple clades of *C. auris*. In subsequent experiments we assessed the impact of the BiNPs on the cell morphology and biofilm structure of *C. auris*.

### 2.1. BiNPs Displayed Strong Antifungal Activity Against all C. auris Strains under Planktonic Growing Conditions

We performed a susceptibility assay to assess the anticandidal activity of the BiNPs against the planktonic stage of multiple strains of *C. auris*, mostly following CLSI methodologies [53] with minor modifications.

We found that most *C. auris* strains exhibited similar susceptibility to the BiNPs, as the MIC values ranged from 1 to 4 µg mL^−1^ (Table 1), with a geometric mean of 2 µg ± 0.7 mL^−1^. The overall anticandidal activity of BiNPs was very similar between the clades, although we noted that two strains from Clade I exhibited slight variations in their susceptibility to BiNPs. The MIC values for the Clade I strains AR no. 0382 and no. 0389 were 1 and 4 µg mL^−1^, respectively; which are within one-step concentration dilution value from the MIC values observed in the other strains (2 µg mL^−1^). As far as the authors know, there are no previous reports on the antifungal activity of BiNPs against *C. auris*. BiNPs displayed strong anticandidal activity against the planktonic stage of all strains, irrespective of their origin, clade, susceptibility profiles against conventional antifungals, and phenotypical and genotypical differences [10,16,17].

Our results also show that the BiNP anticandidal activity under planktonic conditions parallels silver nanoparticles (AgNPs), which usually display MIC values within the 1 to 10 µg mL^−1^ range [36]. This parallelism is relevant because AgNPs are among the most potent antimicrobial nanomaterials [54]. Also, the anticandidal activity of BiNPs is comparable to that of repositionable compounds, such as miltefosine and iodoquinol (MIC = 4 µg mL^−1^), as reported previously by our group [26]. Additionally, the activity of BiNPs matches the proposed activity of the main antifungal drugs. Currently, there are not established antifungal MIC breakpoints for *C. auris*; however, the CDC has proposed the following MIC values to indicate resistance: >32 µg mL^−1^ for fluconazole and >2 µg mL^−1^ for both amphotericin B and caspofungin [16]. Fluconazole is one of the most used antifungal agents against *Candida* infections [21], but *C. auris* is resistant to it; however, amphotericin B and caspofungin display stronger antifungal activity against *C. auris*.

### 2.2. BiNPs Inhibit Biofilm Formation by C. auris

We evaluated the antibiofilm activity of BiNPs during the biofilm formation stage of *C. auris*. As biofilms confer resistance to antifungal drugs and sanitizers, it is clinically relevant to assess the ability of nanoparticles to inhibit biofilm formation. The inhibitory activity of BiNPs on the biofilm was measured using an XTT-reduction assay that measures the metabolic activity of sessile cells within the biofilms, as originally described by our group [55].

**Figure 1:**
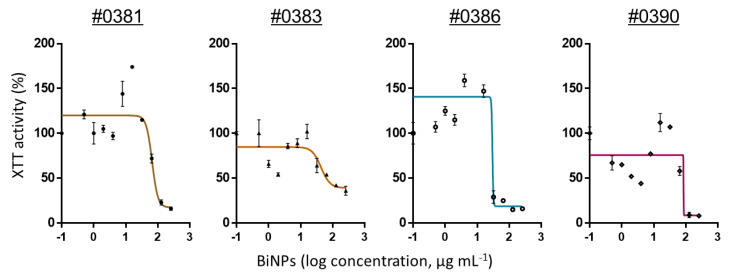
BiNPs reduce the ability of *C. auris* to form biofilms during the biofilm formation stage. The dose–response curves (continuous line) show the effect of the BiNPs (as XTT absorbance readings) during biofilm formation in representative strains from the four clades. The individual points show the average absorbance % over the BiNP concentration, whereas de vertical bars in each point represent the standard deviation (*n* = 6).

**Figure A1:**
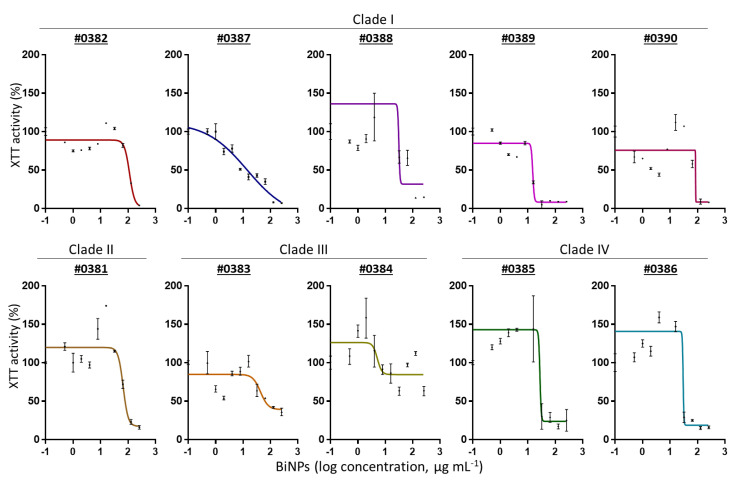
Susceptibility of the multiple *C. auris* strains, clustered by clade, exposed to BiNPs during the biofilm formation stage. The dose–response curves (continuous line) show the effect of the BiNPs (as XTT absorbance readings) during biofilm formation in the multiple strains of *C. auris*, clustered by clade. Within each graph, the individual points show the average absorbance % over the BiNPs concentration, whereas de vertical bars in each point represent the standard deviation (*n* = 6).

BiNPs inhibited the biofilm formation in all *C. auris* strains. The calculated BiNPs IC_50_ values ranged from 5.1 to 113.1 µg mL^−1^, with a geometric mean of 26.8 ± 24.5 µg mL^−1^ (Table 2). The IC_50_ values indicate that each *C. auris* strains had a unique susceptibility to BiNPs, as the biofilm activity was different in each strain. However, the strains from Clade IV were the only ones that showed a consistent behavior to the BiNP treatments. In Figure 1 dose–response curves show the antibiofilm activity against a representative *C. auris* strain from each one of the four clades. The individual dose–response curves for all the *C. auris* AR strains are shown in Figure A1 (Appendix B). It seems that the susceptibility to BiNPs among the different strains is not related to the clade. As *C. auris* is an emergent pathogen, the research regarding their particular physiological traits of each strain is still ongoing, particularly regarding the behavior and differences of the biofilm stages [56,57]. Moreover, the biofilm stage has remained poorly understood up to date, even in thoroughly studied *Candida* species such as *C. albicans* [58].

We noted that the antibiofilm activity of BiNPs was close to the activity reported for other anticandidal agents during the biofilm formation stage. BiNPs displayed lower antibiofilm potency than that of AgNPs against *C. auris* biofilms [29,47]. However, the fact that BiNPs display antibiofilm activity should be considered for further research. Regarding current antifungal drugs, BiNPs display greater antibiofilm potency than fluconazole (IC_50_ > 64 µg mL^−1^), but lower than caspofungin (IC_50_ = 5 to 1 µg mL^−1^) and amphotericin B (IC_100_ = 1 to >8 µg mL^−1^), according to Dekkerová et al., for the AR no. 0383, no. 0386, and no. 0390 *C. auris* strains [59].

An interesting observation is that the biofilm activity –as measured by the XTT absorbance− displayed an irregular behavior in response to the BiNP treatments (Figure A1 (Appendix B)). Some *C. auris* strains exhibited an increase in the biofilm activity—higher than the untreated control—when treated with sublethal concentrations of BiNPs. After the peak of intensity—up to 2.5 times higher than in the control—the activity declined again as the BiNP concentrations increased.

### 2.3. BiNPs Alter the Cellular Morphology and Structure of C. auris Biofilms

To assess the effects BiNPs on the structural organization of *C. auris* biofilms and cell morphology, we analyzed the untreated and the BiNP-treated samples via scanning electron microscopy (SEM). All the strains of *C. auris*, from the four main clades, were exposed to sub-inhibitory concentrations of BiNPs during the biofilm formation phase. Then, we visualized the impact of the nanoparticles on the fungal cell morphology and biofilm structure. As the different strains exhibited variations in their susceptibility to BiNPs, the concentration of BiNPs used for the SEM analysis was selected according to their corresponding calculated IC_50_ values (Table 2) and the experimental concentration from the antibiofilm assays (Section 2.2). Therefore, for each strain, the selected BiNP subinhibitory treatment was the higher experimental concentration of BiNPs closest to the calculated IC_50_ values.

#### 2.3.1. Clade I (South Asia Clade)

**Figure 2:**
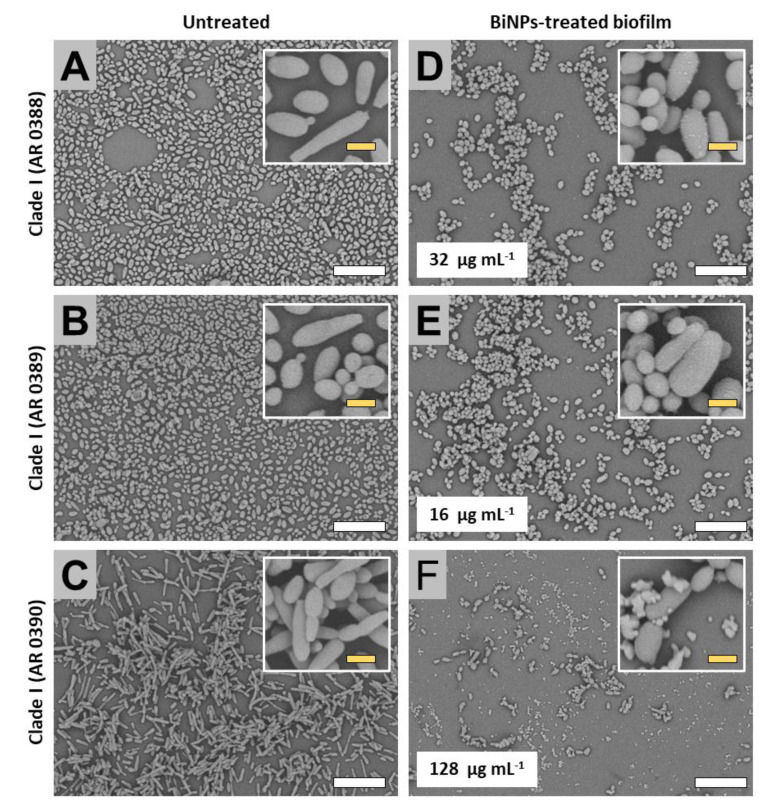
SEM observations on the BiNP antibiofilm activity on the *C. auris* strains from the Clade I (subgroup 1). BiNPs display a reduced biofilm formation—when compared to the untreated control—on the *C. auris* strains no. 0388 (**A**,**D**), no. 0389 (**B**,**E**), and no. 0390 (**C**,**F**). The cell morphology is also affected by the BiNPs (A–F). Scale bar: yellow 2 µm, white = 20 µm.

**Figure 3:**
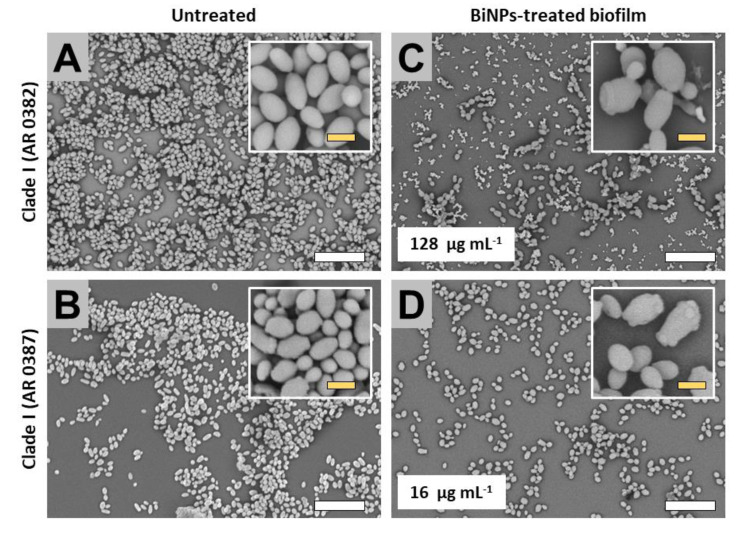
SEM observations on the BiNP antibiofilm activity on the *C. auris* strains from the Clade I (subgroup 2). BiNPs displayed an evident biofilm reduction—when compared to the untreated control—on the *C. auris* strains no. 0382 (**A**,**C**) and no. 0387 (**B**,**D**). The cell morphology also exhibited changes by the BiNPs (A–D). Scale bar: yellow 2 µm, white = 20 µm.

SEM images revealed that the untreated strains form clade I displayed two major structural behaviors. The untreated samples cells first subgroup (AR strains no. 0388, no. 0389, and no. 0390), exhibited both the yeast and pseudohyphae morphologies within the biofilms, whereas the cells from the second subgroup (strains no. 0382 and no. 0387) only displayed the yeast-like shape. The presence of the pseudohyphae-like phenotype has been also described in other works [17]. Subgroup 1: Untreated samples from this subgroup formed biofilms that extensively covered the surface in the bottom of the well (Figure 2A–C), whereas in the BiNP-treated samples, the covered area by the biofilm was noticeably reduced in all strains, regardless of the BiNP concentration in the different samples (from 16 to 128 µg mL^−1^) (Figure 2D–F). The dominant shape for the no. 0390 strain is the pseudohyphae (Figure 2A), whereas, for the no. 0388 and no. 0389 strains, the yeast morphology is the most common (Figure 2B,C). On the BiNP-treated, the presence of the pseudohyphae morphology was reduced in all strains (Figure 2D–F). Subgroup 2: Similar to the subgroup 1, untreated samples also form biofilms that extensively cover the surface in the bottom of the well (Figure 3A,B), whereas the BiNP-treated samples exhibited an evident reduction in all strains, regardless of the BiNPs concentration (16 and 128 µg mL^−1^) (Figure 3C,D). In contrast to the subgroup 1, the cells from the subgroup 2 (strains no. 0382 and no. 0387), only display the yeast-like shape, with no evidence of the pseudohyphae-like shape (Figure 3A,B). The BiNP-treated samples preserved the morphology of the cells, although some alterations on cell size and shape were observed (Figure 3C,D).

#### 2.3.2. Clade II (East Asia Clade)

**Figure 4:**
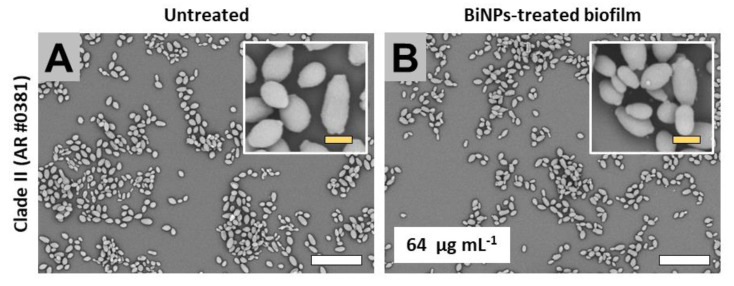
SEM observations on the BiNP antibiofilm activity on the *C. auris* strains from the Clade II. The subinhibitory concentrations of the BiNPs mildly reduced the biofilm formation (**B**) when compared with the control (**A**) on the *C. auris* strains no. 0381. The cell morphology remained mostly unaltered by the BiNP treatment (**A**,**B**). Scale bar: yellow 2 µm, white = 20 µm.

The AR no. 0381 strain is the only one from this clade in the CDC panel. The biofilms from the untreated samples partly covered the bottom of the well (Figure 4A), and the cells display the typical yeast-like morphology (Figure 4A). BiNP-treated samples (64 µg mL^−1^) did not reveal a noticeable reduction in the biofilm formation (Figure 4B); moreover, the cells did not show evident alterations on cell morphology (Figure 4B) when compared with the untreated control.

#### 2.3.3. Clade III (Africa Clade)

**Figure 5:**
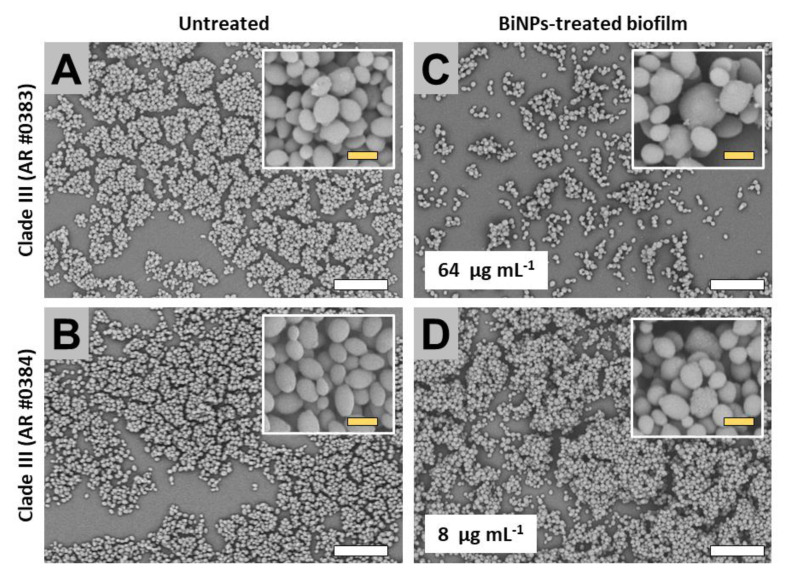
SEM observations on the BiNPs antibiofilm activity on the *C. auris* strains from the Clade III. The BiNP-treated strain displayed a reduced biofilm formation when compared to the untreated control no. 0383 (**A**,**C**), in contrast, the strain no. 0384 biofilms were not noticeably reduced (**B**,**D**). The cell morphology was affected by the BiNPs in both strains, no. 0383 (**A**,**C**) and no. 0384 (**B**,**D**). Scale bar: yellow 2 µm, white = 20 µm.

Untreated samples from the AR no. 0383 and no. 0384 strains formed biofilms that moderately covered the well surface (Figure 5A,B). The cells from both strains exhibited the typical yeast-like shape (Figure 5A,B). Subinhibitory concentrations of the BiNPs inhibited the biofilm formation in the no. 0383 strain, but the lower concentration used for strain no. 0384 (based on initial calculated IC50 values) did not result in significant inhibition of biofilm formation (Figure 5C,D). BiNPs altered the cell morphology in both strains (Figure 5C,D).

#### 2.3.4. Clade IV (South America Clade)

**Figure 6:**
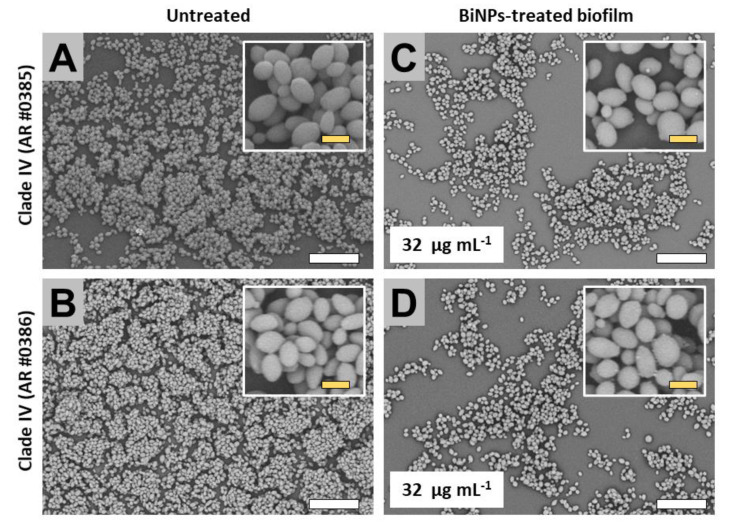
SEM observations on the BiNP antibiofilm activity on the *C. auris* strains from the Clade IV. When compared to the untreated control, BiNP-treated samples exhibited a reduction in the biofilm formation on both strains no. 0385 (**A**,**C**), and no. 0386 (**B**,**D**). No effect on cell morphology was observed on BiNP-treated samples (**B**,**D**). Scale bar: yellow 2 µm, white = 20 µm.

The untreated biofilms from the AR no. 0385 and no. 0386 strains extensively covered the surface of the well (Figure 6A,B). Also, for both strains, cells within the biofilms displayed the typical yeast morphology (Figure 6A,B). The BiNP-treated samples (BiNPs = 32 µg mL^−1^ for both strains) exhibited a visible reduction in the covered area (Figure 6C,D); however, the cell morphology was mostly unaltered by the treatments.

We observed that the untreated samples from the different *C. auris* strains display variations on their phenotypes. This diversity of size and shapes has been described in other works [17,47]. In this work, we expand the current knowledge as we associate the observed specific phenotypes to particular clades.

On the BiNP-treated samples, we observed that nanoparticles altered the shape and size of the yeast cells in some strains. The cause of those alterations is unknown, as this is the first time that the effects of BiNPs are assessed in *C. auris*. Moreover, although the antimicrobial activity of BiNPs and thiolated bismuth complexes have been studied by different groups [60,61,62], their physicochemical interactions between BiNPs and microbial cells remains largely unknown. Bismuth nanoparticles likely exert anticandidal activity by their own, and also may be releasing antimicrobial bismuth ions and thiolated bismuth compounds, as it has been observed in other metallic nanoparticles [44,63]. Moreover, it has been suggested that metal-containing compounds are a promising alternative for developing novel substances with antibiotic properties [12], and the results from different research groups—including ours—increase the evidence for supporting that premise. Nevertheless, the mode of action of BiNPs and the physicochemical interactions within the nanoparticles–cells–biomolecules complex system remains yet to be addressed.

## 3. Materials and Methods 

### 3.1. Material and Strains

Menadione, 2,3-Bis(2-methoxy-4-nitro-5-sulfophenyl)-2H-tetrazolium-5-carboxanilide salt (XTT), and phosphate-buffered saline (PBS), from Sigma-Aldrich (MO). Osmium tetroxide (OsO_4_) and glutaraldehyde were acquired from Ted Pella. 0.74 mM XTT, 10 mM menadione, and 1% OsO_4_ solutions were prepared in Milli Q water.

Nanoantibiotics: We used PVP-coated bismuth nanoparticles (BiNPs) synthesized by a method previously reported by our group [51]. These PVP-BiNPs were produced via a fast, facile, and cost-effective chemical reduction process. Briefly, bismuth nitrate salts were added to a stirring glycine solution, pre-warmed at 75 °C; then pH was raised to 9 using NaOH. After, dimercaptopropanol and PVP solutions were consecutively added to the warm alkaline bismuth solution, still under constant stirring. Finally, NaBH_4_ was added dropwise in two stages. The formation of the BiNPs is rapidly evident by the change of color of the suspension, from yellow to black. The suspension was kept on vigorous stirring for 10 min. This synthesis protocol can be easily replicated in non-specialized facilities, and nanoparticles can be produced in less than 1 h. The obtained BiNPs are small spheroids (average diameter of 8 nm), with a negative surface charge.

Strains: We used 10 different *Candida auris* strains, from the Centers for Disease Control and Prevention (CDC) Antimicrobial Resistance (AR) Isolate Bank stock [64]. The AR strains, organized by clade, are Clade I: South Asia—(AR strains no. 0382, no. 0387, no. 0388, no. 0389, and no. 0390); Clade II: East Asia—(AR strain no. 0381); Clade III: Africa—(AR strains no. 0383 and no. 0384); and Clade IV: South America—(AR strains no. 0385 and no. 0386). Freshly cultured *C. auris* strains were prepared as follows: a loopful of *C. auris* cells from frozen glycerol stocks were subcultured onto yeast extract–peptone–dextrose (YPD) (BD Difco, MD), at 30 °C for 48 h. Then, a couple of *C. auris* colonies were transferred into YPD broth and incubated at 35 °C, overnight, in an orbital shaker. Cells from these fresh cultures were used for the susceptibility assays described in the following sections. 

### 3.2. Susceptibility Tests on Planktonic Cells

To assess the antifungal activity of BiNPs, the CLSI M27 protocol [53] was followed, with slight modifications. Briefly, yeast cells from the overnight cultures were washed twice in PBS and adjusted in RPMI culture media and transferred to a 96 multi-well round-bottom plate. BiNPs were prepared in a two-fold dilution series in RPMI and transferred to the multi-well plates with the yeast cells. The experimental final concentrations of yeast were 2.5 × 10^3^ cells mL^−1^, whereas the BiNPs serial dilutions ranged from 0.5 to 256 µg mL^−1^. Plates with BiNP-treated cells and the controls (untreated cells, BiNPs with no cells, blank RPMI with no cells, and no BiNPs) were cultured for 48 h, at 180 rpm, 35 °C. The Minimal inhibitory concentration (MIC) was set as the concentration where no microbial growth was observed visually, according to the guidelines of the M27 protocol from the CLSI. 

### 3.3. Antibiofilm Activity Assays

The inhibitory effect of BiNPs on the biofilm formation stage of *C. auris* was determined using a method previously reported by our group [55], with minor modifications. Briefly, the yeast cells from the overnight cultures were washed twice in PBS and the cells were adjusted in RPMI and transferred to flat-bottom 96-multiwell plates. BiNPs were prepared in a two-fold dilution series, then transferred to the multi-well plates with the yeast cells. The experimental final concentration for the biofilm assays was 1 × 10^6^ cells mL^−1^; whereas the BiNP serial dilutions ranged from 0.5 to 256 µg mL^−1^. The multi-well plates with the BiNP-treated yeast cells and the controls (untreated cells, BiNPs with no cells, blank RPMI with no cells, and no BiNPs) were cultured stationery, at 37 °C for 24 h, for inducing the biofilm formation phase.

To assess the antibiofilm effect of BiNPs, the XTT absorbance was used as a measure of biofilm activity [55], as follows: post-incubation, biofilms were washed twice with PBS and 100 µL of XTT/menadione solution were added to the plates with the treated and the controls. Immediately, the plates were protected from light and incubated for 2 h at 37 °C. The absorbance of XTT was measured at λ = 490 nm in a Benchmark Microplate Reader (Bio-Rad Inc). From the absorbance readings, dose–response curves were obtained to calculate the IC_50_, set as the concentration of BiNPs required for reducing the biofilm activity by 50%. The IC_50_ was calculated by fitting the normalized results to the variable slope Hill equation (to assess the nonlinear dose–response relationship), in the software Prism 8 (GraphPad Software, Inc.).

To ensure the experimental reproducibility, the anticandidal activity of BiNPs was evaluated using different rounds of nanoparticle syntheses, using three independent replicates of the 96-multiwell plates, with two replicates of the treatments within each multiwell plate (*n* = 6, for each strain), for both the planktonic and the biofilm stages. 

### 3.4. Ultrastructural Analysis

The effect of the BiNPs on the structure of the *C. auris* biofilms was evaluated in the inhibition of biofilm formation experiments via scanning electron microscopy (SEM). Briefly, biofilms were treated with subinhibitory concentrations of BiNPs, according to the corresponding calculated IC_50_ values for each *C. auris* strain. After incubation, the biofilm samples were washed twice with PBS, then fixed with 2% glutaraldehyde for 3 h, and then stained with 1% osmium tetroxide for 30 min. Then, the samples were dehydrated using an ascending concentration series of ethanol, from 30% to 100%. Then, ethanol was completely removed, and the samples were left to dry overnight. The dehydrated samples were coated with gold in a sputter coater SC7620 (Quorum Technologies). To form a uniform and thick gold layer over the samples, the current was set at 25 milliamperes for 3 min. Finally, the samples coated with gold were observed in a TM4000Plus Scanning Electron Microscope (Hitachi Inc.), using the 500× and 2500× magnification, with a voltage of 10 KeV.

## 4. Conclusions

Overall, our results seem to indicate that bismuth nanoparticles display a strong anticandidal activity against all the multiple *C. auris* CDC AR strains when tested under planktonic conditions. However, the BiNPs displayed much more modest antibiofilm activity, with also more accused differences among the different strains, which were not necessarily related to their clade. Despite this lower activity, treatment with BiNPs affected the biofilm structure and, in some instances, the cell morphology of cells within biofilms. Our results seem to indicate that the broad anticandidal activity of bismuth nanoparticles may contribute to reducing the spread of the multidrug-resistant *C. auris* strains in the healthcare-related facilities. Finally, more studies regarding the antimicrobial properties of BiNPs, against different pathogens, will contribute to expanding their future potential applications.

## Figures and Tables

**Table 1:** Planktonic MIC values (µg mL^−1^) of BiNPs against *C. auris* strains from the different clades.

Clade	Strains	MIC	Geometric Mean
I	0382	1	2
	0387	2	
	0388	2	
	0389	4	
	0390	2	
II	0381	2	2
III	0383	2	2
	0384	2	
IV	0385	2	2
	0386	2	

**Table 2:** Calculated IC_50_ (µg mL^−1^) for the biofilm inhibitory activity of BiNPs against *C. auris* strains from the different clades.

Clade	Strains	IC_50_	Geometric Mean
I	0382	113.1	27.5
	0387	14.1	
	0388	31.4	
	0389	15.2	
	0390	85.2	
II	0381	66.5	66.5
III	0383	43.6	14.9
	0384	5.1	
IV	0385	27.7	28.8
	0386	29.9	

## Appendix A

Planktonic stage: Is the state in which the cells are free-living, acting as independent organisms.

Biofilm stage: Is the state when the cells are attached to a surface, forming multicellular communities, protected by an extracellular matrix [65]. Biofilms can transition between both the planktonic and the biofilms stages. Traditionally, it is considered that biofilms undergo a maturity process: from the biofilm formation (adhesion stage) to the mature biofilms (development and maturation stages).

Yeast phenotype: It is the typical single-cell oval-shape structure seen for most *Candida* species. However, there are several morphological variations across different *Candida* strains and species, resulting in shapes such as ellipsoidal, elongated, and rectangular with smooth rounded ends, among others.

Pseudohyphae phenotype: Some pleiomorphic fungus, such as *C. albicans*, can undergo a morphogenesis process for transitioning (forth and back) to a pseudohyphal or hyphal phenotype. [66,67]. Pseudohyphae displays a multicellular organization, as the daughter cells bud elongates and remains attached to the mother cell, after septum formation, forming strings of connected budding cells. These filaments can grow so long that they resemble hyphae. Some strains of *C. auris* can form elongated cells that resemble pseudohyphae [9].

Hyphae phenotype: The hyphal phenotype is characterized by the extreme elongation of the fungal cells, leading to the formation of long germ tubes that are usually narrower than pseudohyphal cells. The initial germ tubes do not display apparent septa [66], and they exhibit polarized growth. The ‘true’ hyphal stage has not been reported in *C. auris*.
